# The association of cadmium and lead exposures with red cell distribution width

**DOI:** 10.1371/journal.pone.0245173

**Published:** 2021-01-11

**Authors:** Junenette L. Peters, Melissa J. Perry, Eileen McNeely, Robert O. Wright, Wendy Heiger-Bernays, Jennifer Weuve

**Affiliations:** 1 Department of Environmental Health, Boston University School of Public Health, Boston, Massachusetts, United States of America; 2 Department of Environmental and Occupational Health, George Washington University, Washington, District of Columbia, United States of America; 3 Department of Environmental Health, Harvard T.H. Chan School of Public Health, Boston, Massachusetts, United States of America; 4 Icahn School of Medicine, Mount Sinai Hospital, New York, New York, United States of America; 5 Department of Epidemiology, Boston University School of Public Health, Boston, Massachusetts, United States of America; Stony Brook University, Graduate Program in Public Health, UNITED STATES

## Abstract

Elevated red blood cell distribution width (RDW), traditionally an indicator of anemia, has now been recognized as a risk marker for cardiovascular disease incidence and mortality. Experimental and acute exposure studies suggest that cadmium and lead individually affect red blood cell production; however, associations between environmental exposures and RDW have not been explored. We evaluated relationships of environmental cadmium and lead exposures to RDW. We used data from 24,607 participants aged ≥20 years in the National Health and Nutrition Examination Survey (2003–2016) with information on blood concentrations of cadmium and lead, RDW and socio-demographic factors. In models adjusted for age, sex, race/ethnicity, education, poverty income ratio, BMI, alcohol consumption, smoking status and serum cotinine, RDW was increasingly elevated across progressively higher quartiles of blood cadmium concentration. A doubling of cadmium concentration was associated with 0.16 higher RDW (95% CI: 0.14, 0.18) and a doubling of lead concentration with 0.04 higher RDW (95% CI: 0.01, 0.06). Also, higher cadmium and lead concentrations were associated with increased odds of high RDW (RDW>14.8%). The associations were more pronounced in women and those with low-to-normal mean corpuscular volume (MCV) and held even after controlling for iron, folate or vitamin B12 deficiencies. In analysis including both metals, cadmium remained associated with RDW, whereas the corresponding association for lead was substantially attenuated. In this general population sample, blood cadmium and lead exposures were positively associated with RDW. The associations may indicate hemolytic or erythropoietic mechanisms by which exposure increases mortality risk.

## Introduction

Evidence suggests that the heavy metals cadmium and lead interfere with a number of functions of the erythropoietic system, the processes that make red blood cells. In addition, both cadmium and lead are highly bound to intracellular and membrane proteins in the red blood cell [1, 2], complicating analysis of their effects on erythropoiesis. Both metals are also toxic to the kidney [3], which is the site of erythropoietin synthesis, the primary hormone that stimulates red blood cell generation in the bone marrow [4]. The proposed mechanisms by which these metals affect erythropoiesis include disruption of heme synthesis, iron accumulation and erythropoietin (EPO) production. In experimental and acute exposure studies, cadmium and lead exposures were associated with reductions in the blood’s oxygen-carrying capacity and disruption to the process of red blood cell production (erythropoiesis) [5–7]. Mechanistic studies also demonstrated the ability of cadmium and lead to prevent the absorption of iron contributing to anemia [8]. Lead poisoning has been related to abnormalities of erythrocyte shape, porphyrin and normoblasts, as well as basophilic stippling [9, 10]. Cadmium appears to reduce gastrointestinal absorption of iron, but there are conflicting results for oral cadmium exposure and anemia. Cadmium accumulates in EPO-producing organs such as the kidney and has been shown to affect EPO synthesis in a dose-dependent manner [7]. EPO is a key hormone in the regulation of erythropoiesis. In human chronic cadmium intoxication (e.g., Itai-itai disease), EPO is not up-regulated as expected given severe anemia [11].

The red blood cell distribution width (RDW) is routinely provided as part of a complete blood count (CBC). It is a quantitative measure of the consistency in size of circulating erythrocytes [12] such that higher RDW reflects greater variability in the size (volume) of red blood cells (RBCs) (the “width” in RDW refers to the width of the distribution curve, not to the cell size). RDW is an indicator of several dysfunctions, such as increased or decreased red cell destruction or production or nutrient deficiencies at the extremes. Immature red blood cells, referred to as reticulocytes, retain a distinct appearance and are larger than mature red blood cells. Thus, RDW would increase with higher presence of reticulocytes when bone marrow is actively generating RBC, which can occur as a result of hemolysis [13]. Hemolysis can occur secondary to oxidative stress because of the sensitivity of RBCs to inflammation [14, 15]. A “high normal” RDW can serve as an integrative measure of subclinical systemic inflammation and other systemic dysfunction [16] such as oxidative stress and hemolysis [17]. Higher RDW has now been associated with cardiovascular morbidity and all-cause mortality in non-patient (the general) population [18–25], and very recently with mortality with severe acute respiratory syndrome coronavirus 2 (SARS-CoV-2) infection [26]. RDW has been proposed in the prognosis of disease and mortality [27–31].

Given the relation of cadmium and lead exposures to anemia in intoxication and experimental studies, and the connections of these two exposures and RDW to CVD outcomes, we evaluated the relation of environmental cadmium and lead exposures to RDW. We also assessed these associations in the context of mean corpuscular volume (MCV) and evaluated the degree to which they differed between men and women. We further sought to test how robust the cadmium analysis was to confounding by cigarette smoking.

## Materials and methods

The National Health and Nutrition Examination Survey (NHANES) is a program of studies that evaluates the health and nutritional status of the noninstitutionalized United States (U.S.) civilian population and provides the data as public-use files. The studies were approved by the National Center for Health Statistics (NCHS) Research Ethics Review Board; NCHS obtained written informed consent from all participants.

Participants for this analysis were part of NHANES survey cycles 2003–2004, 2005–2006, 2007–2008, 2009–2010, 2011–2012, 2013–2014, and 2015–2016. The final data set was accessed in September 2019. A total of 29,700 non-pregnant participants ≥20 years of age had RDW, blood cadmium and blood lead concentrations. Our analyses included the 24,608 who additionally had measures of demographic, behavioral and health factors. S1 Fig contains a flow diagram outlining the study population selection.

### Laboratory procedures

The CBC, which included RDW (%), was performed using the Beckman Coulter MAXM Instrument (Beckman Coulter Corporation, Miami, FL). RDW is the coefficient of variation of RBC size, expressed as a percentage, specifically, the standard deviation of RBC volume divided by the mean corpuscular volume (MCV). MCV was also measured as part of the CBC and is often used in clinical settings in combination with RDW.

The concentrations of cadmium and lead were quantified in whole blood using dynamic reaction cell for inductively coupled plasma-mass spectrometry (ICP-DRC-MS) technology. The limit of detection for cadmium was 0.89 nmol/L (0.10 μg/L) and for lead was 0.01 μmol/L (0.25 μg/dL) for 2003–2012 and 0.003 μmol/L (0.07 μg/dL) for 2013–2016. NHANES assigned samples with concentrations below the lower limit of detection (LLOD), a value of the detection limit divided by the square root of two. Nearly one in five participants (17%) had blood cadmium concentrations below the LLOD (0.89 nmol/L), whereas only 0.26% had blood lead concentrations below the LLOD (0.01 μmol/L).

Whole blood hemoglobin and serum cotinine were measured; serum folate, vitamin B12 and ferritin were also measured for specific years. Detailed information of all laboratory procedures is available at http://www.cdc.gov/nchs/about/major/nhanes/datalink.htm.

### Statistical analysis

We estimated the mean difference in RDW across blood concentrations of lead and cadmium using multivariable-adjusted linear regression models adjusting for variables determined a priori: age, sex, race/ethnicity, education, poverty-income ratio, body mass index (BMI), alcohol consumption, smoking status, serum cotinine concentration, and survey cycle (main model). In particular, the variable race/ethnicity as defined by NHANES was included to account for variations in cadmium levels, RDW trends and anemias by this construct, which may be driven by cultural diversity (e.g., diet), genetics (e.g., sickle cell anemia), or discrimination [32–35]. Iron deficiency anemia is one mechanism by which RDW is altered [36]; therefore, to determine the relationship between RDW and cadmium and lead independent of this deficiency, we also estimated the mean difference in RDW across lead and cadmium concentrations further adjusting the main model for iron-deficiency. Socio-demographic and health characteristics were parameterized as outlined in Table 1 except for age and BMI, which were modeled as continuous variables, and serum cotinine concentration, which was modeled as a log-transformed continuous variable. Iron deficiency was defined as hemoglobin <13 g/dL in men and hemoglobin <12 g/dL in women [37]. In addition to fitting separate models for blood cadmium and lead, we also fitted models including both exposures.

**Table 1:** Participant characteristics.

		Geometric mean concentration (SE)^a^	
Variable	N	Blood Cadmium, nmol/L	Blood Lead, μmol/L
Age (years)			
20–39	8068	2.6 (0.01)	0.04 (0.01)
40–59	8084	3.3 (0.01)	0.07 (0.01)
≥ 60	8456	3.6 (0.01)	0.08 (0.01)
Sex			
Women	12147	3.4 (0.01)	0.05 (0.01)
Men	12461	2.8 (0.01)	0.07 (0.01)
Race/Ethnicity			
Mexican	3978	2.6 (0.02)	0.06 (0.03)
Black	4959	3.5 (0.02)	0.06 (0.02)
Other Hispanic	1969	2.7 (0.03)	0.05 (0.03)
Other Race	1856	3.9 (0.03)	0.06 (0.02)
White	11846	3.1 (0.01)	0.06 (0.01)
BMI (kg/m^2^)^b^			
<25	7214	3.5 (0.02)	0.06 (0.01)
25–29	8334	3.0 (0.02)	0.06 (0.01)
≥ 30	9060	2.9 (0.01)	0.06 (0.01)
Education			
< High School	6276	4.1 (0.02)	0.07 (0.02)
High School/GED^c^	5743	3.5 (0.02)	0.06 (0.02)
> High School	12589	2.7 (0.01)	0.05 (0.01)
Poverty^d^			
Yes	4947	3.9 (0.03)	0.06 (0.02)
No	19661	3.0 (0.01)	0.06 (0.01)
Smoke			
Never	13031	2.1 (0.01)	0.05 (0.01)
Former	6256	2.9 (0.01)	0.07 (0.01)
Active	5320	8.1 (0.02)	0.07 (0.02)
Alcohol (drinks/day)			
<1	17621	3.1 (0.01)	0.06 (0.01)
1 to 4	5541	2.9 (0.02)	0.07 (0.01)
≥ 5	1446	4.1 (0.03)	0.07 (0.02)
Iron Deficiency^e^			
Yes	2145	3.3 (0.02)	0.06 (0.02)
No	22463	3.1 (0.01)	0.06 (0.01)
Mean Corpuscular Volume (fL/red cell)			
<80	1240	3.4 (0.03)	0.05 (0.02)
80–95	20138	2.9 (0.01)	0.06 (0.01)
>95	3230	4.3 (0.02)	0.07 (0.02)
Red Cell Distribution Width (quartiles, %)			
1 (10.7– 12.2)	5682	2.8 (0.02)	0.06 (0.01)
2 (12.3–12.6)	6341	3.0 (0.01)	0.06 (0.01)
3 (12.7–13.3)	6784	3.1 (0.02)	0.06 (0.01)
4 (13.4–37.8)	5801	3.7 (0.02)	0.06 (0.01)

^a^Geometric mean blood concentrations of cadmium and lead (and standard errors of the mean [SE]) by participant characteristics using the National Health and Nutrition Examination Survey (NHANES) sample weighting.

^b^BMI is body mass index calculated as weight (kg) divided by height squared (m^2^).

^c^GED is general education development or high school equivalency certificate.

^d^Poverty is defined as having poverty income ratio <1.00, where poverty income ratio is the ratio of the family income to the appropriate poverty threshold.

^e^Iron deficiency is defined as hemoglobin <13 g/dL in men and hemoglobin <12 g/dL in women.

For our primary analyses, we performed linear regressions for the main model and model further adjusting for iron deficiency assessing differences in RDW 1) by quartiles of blood cadmium and lead and 2) per doubling of cadmium and lead concentrations (e.g., β [ln (Cd)] x ln [2]). We modeled cadmium and lead in quartiles to assess the monotonicity of associations with RDW and as log-transformed continuous variables to reduce the influence of potential outliers. We also performed main model and added iron deficiency model logistic regressions of the odds of high RDW for two-fold increases in continuous cadmium and lead. RDW was categorized as high (>14.8%) as defined in previous studies relating RDW to health outcomes [38–40].

We also performed stratified analyses. We first investigated the association of blood cadmium and lead with RDW, stratified by sex [33]. We second evaluated the associations with strata of MCV, further stratified by sex. MCV was categorized as low (<80 fL/red cell), normal (80–95 fL/red cell) and high (>95 fL/red cell). We quantified differences in the metal-RDW association across strata of sex and MCV status by including cross-products (e.g., centered blood cadmium*sex) in models with continuous RDW.

In addition, we performed sensitivity analyses. Iron deficiency can be determined using serum ferritin (<30 μg/L) [37]; however, NHANES only measured serum ferritin in adults in cycles 2003–2004, 2005–2006, 2007–2008, 2009–2010 and 2015–2016, and in the later four cycles, only in females 20–49 (N = 4,519). Thus, our first sensitivity analysis was to evaluate the association of blood cadmium and lead with RDW on females 20–49 years of age using the definition of iron deficiency based on serum ferritin to compare with results in that same group using the definition based on hemoglobin. Vitamin B12/folate deficiency is also related to changes RDW [41]. However, information on B12 deficiency was not available in cycles 2007–2008, 2009–2010, and 2015–2016. Therefore, the second sensitivity analysis involved further controlling for folate and vitamin B12 deficiencies in models controlling for iron deficiency (N = 13,372). To test the robustness of the cadmium analysis to confounding by cigarette smoking, the third sensitivity analysis estimated the associations of cadmium biomarkers separately for never smokers (reported never smoking and had low cotinine levels [<1 ng/mL]) and ever smokers (reported being past or current smokers or had high cotinine [≥1 ng/mL])

All analyses accounted for the complex, multistage, probability sampling method of NHANES. Analyses were performed using SAS version 9.4 (SAS Institute Inc., NC).

## Results

The median (standard error; SE) RDW among the participants in our analysis was 12.6% (0.01%) with a 25^th^ percentile of 12.2% (0.01%) and 75^th^ percentile of 13.3% (0.02%). The clinically normal range for RDW is 11.8–14.8% [42]; 1,688 (6.9%) of participants had RDWs above 14.8%. The geometric mean (GM) and (SE) for blood cadmium concentration was 3.1 nmol/L (0.01) and for blood lead, 0.06 μmol/L (0.01). The Spearman correlation between blood cadmium and blood lead was 0.35.

In unadjusted analyses, blood cadmium concentration was higher among participants who were: older, female, non-Hispanic black and other race, current smokers, had less education, higher poverty-to-income ratio, lower BMI, and higher alcohol consumption (Table 1). Unadjusted associations of blood lead concentration with these variables were generally weaker but similar, with the exception of sex; blood lead concentration was higher among male participants.

### Associations of cadmium and lead with RDW

**Fig 1:**
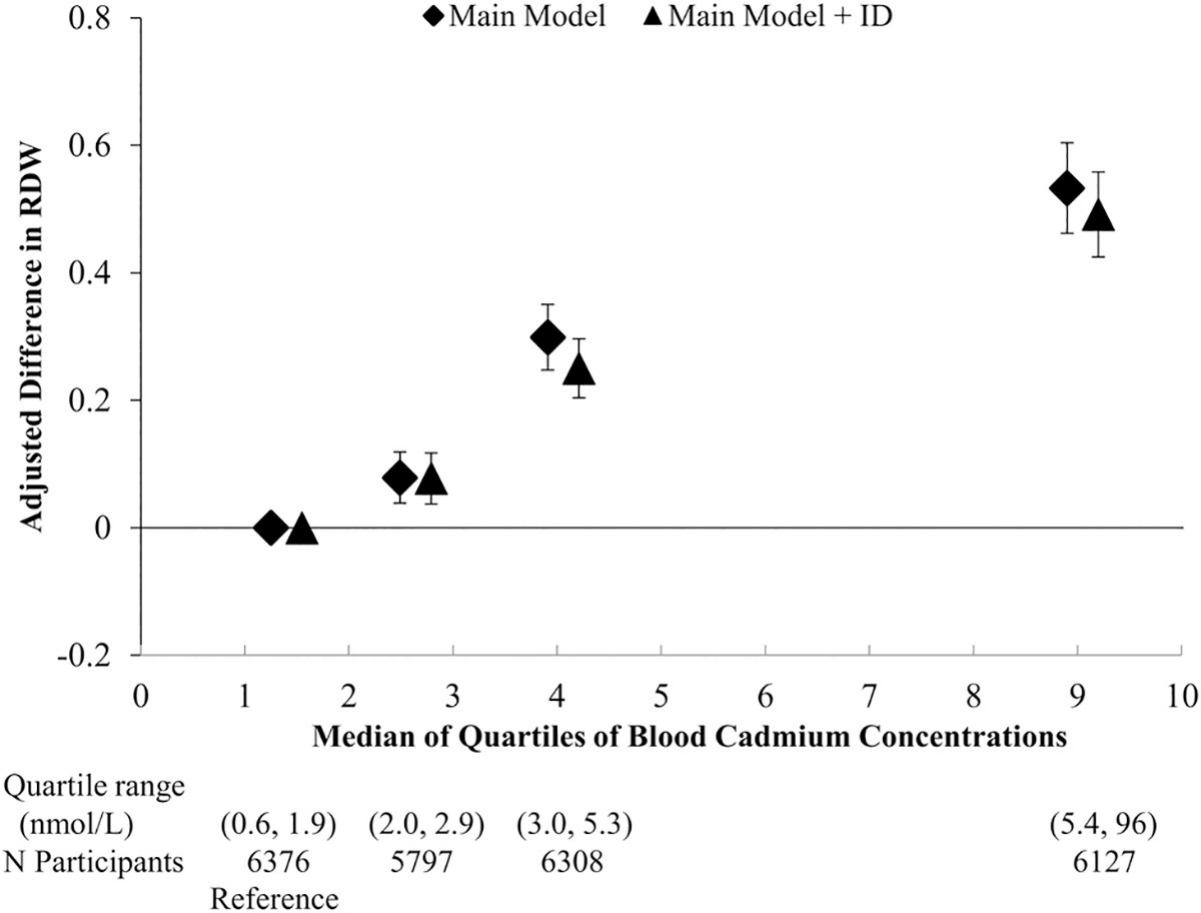
The relation of quartiles of blood cadmium to red cell distribution (RDW). Adjusted for age, sex, race/ethnicity, education, poverty income ratio, body mass index, alcohol consumption, smoking status, serum cotinine, and survey cycle (main model) and in addition, iron deficiency (ID).

Mean RDW was progressively higher with higher quartile of blood cadmium, after adjustment for age, sex, race/ethnicity, education, poverty income ratio, BMI, alcohol consumption, smoking status, serum cotinine and survey cycle and also after further adjustment for iron deficiency (Fig 1). Considering blood cadmium concentration as a log-transformed continuous variable, a doubling of cadmium concentration was associated with 0.16 higher RDW (Table 2; main model). Additional adjustment for iron deficiency attenuated these results only slightly (difference in RDW per doubling of Cd concentration = 0.15; also see Table 2). A two-fold elevation in cadmium concentration corresponded to 67% increased odds of high RDW (RDW > 14.8%) (95 CI: 51%, 84%), an association that was strengthened with further adjustment for iron deficiency (OR = 1.73 [95% CI: 1.56, 1.92]) (S1 Table).

**Table 2:** Multivariable-adjusted differences in red cell distribution width (RDW) for two-fold increases in blood cadmium and blood lead exposures.

			Mean difference in RDW, % (95% CI) per doubling of blood metal concentration			
		N	Cadmium		Lead	
All, main model^a^		24,608	0.16	(0.14, 0.18)	0.04	(0.01, 0.06)
	Further adjusted for iron deficiency (ID) ^b^		0.15	(0.13, 0.17)	0.05	(0.03, 0.08)
All, main model, adjusted for the other metal^c^		24,608	0.16	(0.14, 0.18)	0.00	(-0.02, 0.03)
	Further adjusted for ID		0.15	(0.13, 0.17)	0.02	(0.00, 0.05)
Females, main model		12,147	0.24	(0.20, 0.28)	0.06	(0.02, 0.10)
	Further adjusted for ID		0.22	(0.18, 0.25)	0.09	(0.04, 0.13)
Males, main model		12,461	0.08	(0.06, 0.10)	0.02	(0.00, 0.05)
	Further adjusted for ID		0.09	(0.06, 0.11)	0.03	(0.01, 0.06)
Normal Mean Corpuscular Volume (MCV), main model^d^		20,138	0.11	(0.09, 0.13)	0.03	(0.01, 0.05)
	Further adjusted for ID		0.11	(0.09, 0.13)	0.04	(0.02, 0.06)
Low MCV, main model		1,240	0.71	(0.67, 0.75)	0.03	(0.01, 0.05)
	Further adjusted for ID		0.57	(0.54, 0.60)	0.12	(0.09, 0.16)
High MCV, main model		3,229	0.07	(0.01, 0.14)	0.02	(-0.05, 0.08)
	Further adjusted for ID		0.09	(0.03, 0.15)	0.04	(-0.02, 0.11)
Ever smoker, main model		11,576	0.10	(0.08, 0.12)	0.06	(0.03. 0.09)
	Further adjusted for ID		0.12	(0.10, 0.13)	0.08	(0.05, 0.10)
Never smoker, main model		13,031	0.24	(0.20, 0.28)	0.04	(0.00, 0.07)
	Further adjusted for ID		0.21	(0.18, 0.25)	0.06	(0.02, 0.09)

Multivariable-adjusted beta estimates for RDW for two-fold increases in blood cadmium and blood lead exposures, analyzed separately and together, for all adults and stratified by categories of sex, MCV and smoking status.

^a^Adjusted for age, sex, race/ethnicity, education, poverty income ratio, body mass index, alcohol consumption, smoking status, serum cotinine, and survey cycle.

^b^Adjusted for all variables in the multivariable-adjusted main model, and in addition, iron deficiency.

^c^Model including both blood cadmium and blood lead exposures.

^d^Normal MCV defined as 80–95 fL/red cell, low MCV as <80 fL/red cell, and high MCV as >95 fL/red cell.

**Fig 2:**
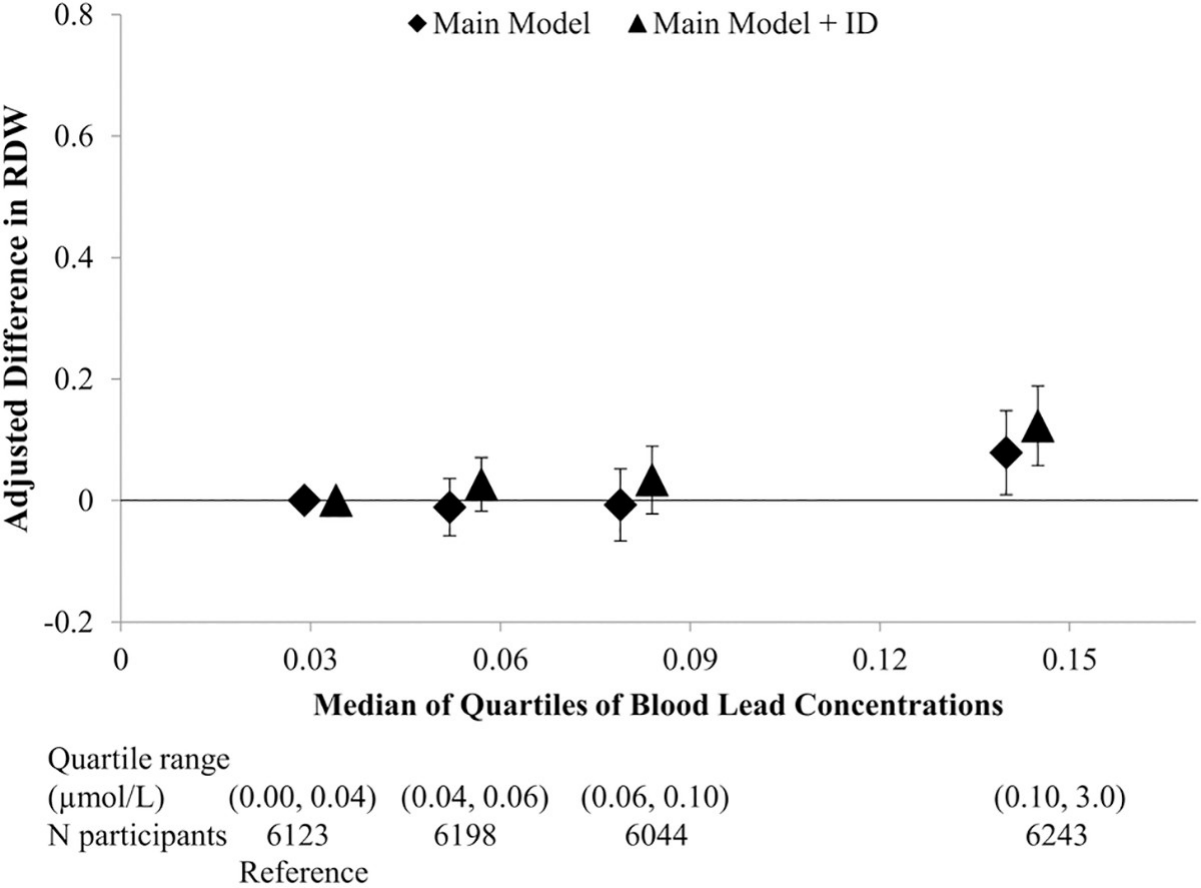
The relation of quartiles of blood lead to red cell distribution (RDW). Adjusted for age, sex, race/ethnicity, education, poverty income ratio, body mass index, alcohol consumption, smoking status, serum cotinine, and survey cycle (main model) and in addition, iron deficiency (ID).

By contrast, RDW did not follow a clear gradient with increasingly higher quartile of blood lead concentration (Fig 2). Adjusting for variables in the main model, RDW was markedly higher only among those in the highest blood lead quartile (range = 0.10–3.0 μmol/L) compared with those in the lowest quartile (range = 0.002–0.04 μmol/L) (difference = 0.08 [(95% CI: 0.01, 0.15]). With further adjustment for iron deficiency, the association to RDW between the highest and lowest blood lead quartile was strengthened (0.12 [(95% CI: 0.06, 0.19]). A two-fold elevation in continuous lead concentration was associated with a 0.04 increase in RDW in the main model, and with a 0.05 increase in RDW in the model further adjusting for iron deficiency (Table 2). A two-fold elevation in lead concentration corresponded to 10% increased odds for high RDW (95% CI: 0.2%, 21%), a relationship that was strengthened with additional adjustment for iron deficiency (OR = 1.21 [95%: 1.09, 1.34]) (S1 Table).

Analyzing blood cadmium and lead together, in the main model, a two-fold elevation in cadmium concentration was associated with a 0.16 increase in RDW and a two-fold elevation in lead concentration with a 0.01 increase in RDW (Table 2). In the model further adjusted for iron deficiency, a two-fold elevation in cadmium concentration was associated with a 0.15 increase in RDW and a two-fold elevation in lead concentration with a 0.02 increase in RDW (Table 2). In addition, cadmium remained a strong predictor of high RDW. In analysis with a multiplicative term of cadmium times high lead concentration (highest lead quartile; range = 0.10–3.0 μmol/L), those with high lead had a difference of 0.04 (95% CI: 0.02, 0.07) for a two-fold increase in cadmium. Similarly, analysis with a multiplicative term of lead times high cadmium (highest cadmium quartile; range = 5.4–96 nmol/L), those with high cadmium had a difference in RDW of 0.02 (95% CI: 0.01, 0.03) with a two-fold increase in lead.

### Stratified analyses

#### Associations by sex

In stratified analyses, the relationship of blood cadmium to RDW was stronger in females than in males (Table 2). In analysis including a multiplicative term for effect modification, females had a difference in RDW of 0.05 (95% CI: 0.03, 0.08) for a two-fold elevation in cadmium in the fully adjusted model (main model further adjusting for iron deficiency). For lead, the relationship was also stronger in females than in males in the fully-adjusted model–a difference in RDW of 0.01 (95% CI: -0.01, 0.03) in females. The relationship of cadmium and lead to high RDW was stronger in females than in males.

#### Associations by mean corpuscular volume

Table 2 shows the relationship of RDW for a two-fold increase in blood cadmium and lead stratified by categories of MVC (normal, low and high). Those with low MCV had a difference in RDW of 0.45 (95% CI: 0.32, 0.59) per two-fold increase in cadmium compared to those with normal MCV and those with high MCV had a difference in RDW of 0.02 (95% CI: -0.02, 0.05) per two-fold increase in cadmium compared with those with normal MCV. In general, a positive effect of cadmium on RDW was more pronounced in those with low MCV and particularly in women with low MCV.

For lead, in continuous analysis of the fully adjusted model, those with low MCV had a difference in RDW of 0.02 (95% CI: -0.09, 0.04) per two-fold increase in lead compared to those with normal MCV and those with high MCV a difference in RDW of 0.04 (95% CI: 0.02, 0.05) per two-fold increase in lead. Odds ratios for high RDW in relation to lead were more pronounced for women with normal and low MCV.

#### Association by smoking status

The association between cadmium and RDW was greater among never smokers (Table 2). In the fully adjusted model, the difference in effect on RDW per two-fold increase in cadmium for never smokers compared to ever smokers was 0.05 (95% CI: 0.02, 0.08). The odds ratio for high RDW for never smokers was 2.20 (95% CI: 1.92, 2.52) and for ever smokers 1.43 (95% CI: 1.29, 1.59). Of note, 26% of never smokers had cadmium levels below LLOD compared to 8% of ever smokers. Fifty-eight percent of never smokers and 40% of ever smokers were female. In model with all interaction terms (i.e., cadmium*sex, cadmium*MCV, and cadmium*ever smoker), the interactions held by sex and MCV for the continuous RDW. The association between lead and RDW was similar among never smokers and ever smokers (Table 2).

#### Sensitivity analyses

In fully adjusted analysis, in women ages 20–49 years controlling for iron deficiency categorized using ferritin, there was a similar positive relationship between blood cadmium concentration and an increase in RDW and high RDW; for example, a two-fold increase in blood cadmium concentration corresponded to a 0.11 (95% CI: 0.06, 0.16) increase in RDW using ferritin and 0.19 (0.14, 0.24) using hemoglobin. In similar analysis with lead as the predictor, we also found a positive relationship between lead and an increase in RDW and high RDW; for example, a two-fold increase in blood lead concentration corresponded to a 0.14 (95% CI: 0.08, 0.21) increase in RDW using ferritin and 0.16 (0.10, 0.23) using hemoglobin.

In the fully adjusted analysis additionally controlling for B12 and folate, there was a positive relationship between blood cadmium and RDW. A two-fold increase in blood cadmium concentration corresponded to a 0.15 increase in RDW (95% CI: 0.12, 0.17). A two-fold increase in blood lead concentration to a 0.02 increase in RDW (95% CI: 0.01, 0.03).

## Discussion

This population-based study provides evidence that environmental cadmium and lead exposures are associated with elevated RDW. These findings have potential implications given continuing reports of associations between elevated RDW and mortality in CVD patients and in the general population. We found that the associations of cadmium and lead with RDW were independent of socio-demographic characteristics, health behavior and nutritional deficiencies. The association was especially strong in relation to cadmium exposure. Furthermore, the relations of cadmium and of lead to RDW were more pronounced among women.

In the U.S., major sources of cadmium exposure include emissions from industrial activity and waste management operations, consumption of foods such as leafy greens, grains, peanuts, soybeans and organ meats, and exposure to cigarette smoke [6, 43]. Lead exposure has significantly declined in the U.S. following the phase out of lead in gasoline and paint in the 1970s and 1980s. However, because in adults, approximately 95% of lead in the body is stored in bone with a half-life of years to decade, even with the decline in sources of environmental lead, lead released from bone stores to blood and soft tissue can act as an ongoing source [44, 45]. Current exposure to lead may be from paint in old houses, lead pipes, food containers such as improperly glazed ceramic dishes, hazardous waste sites, busy highways once contaminated, and cigarette smoke [5].

Our observation that environmental exposure to cadmium, and to a lesser extent lead, are associated with elevated RDW has implications for cadmium’s impact on health given increasing literature supporting RDW as a marker of mortality [27–30]. Previous studies have found that a percentage point increase in RDW has been associated with a 23% increase in mortality risk in the general population [46]. Our observation also has implications related to the global impact of anemia insofar as anemia is characterized by increased RDW. Anemia was observed in the case of a mass cadmium poisoning in the Toyama Prefecture, Japan (i.e., the Itai-itai disease) and among industrial exposed workers [47]. The anemia observed in Itai-itai disease patients was believed to be primarily the result of impaired production of EPO [48]. Indeed, cadmium accumulates in the liver and kidney, both EPO producing organs [49].

Lead can both inhibit heme synthesis and shorten the lifespan of erythrocytes [5, 50]. Lead inhibits delta aminolevulinic acid dehydratase (δ-ALAD) of erythrocytes and ferrochelatase, which disrupts heme synthesis [51, 52]. Inhibition of ferrochelatase is probably a result of the action of lead on bone marrow erythroblasts which contain mitochondria and synthesize heme [51]. Lead can also affect EPO production thereby affecting the maturation of blood progenitor cells [5]. The inhibition of heme synthesis may be secondary to RBC destruction (hemolysis). Lead poisoning has been associated with hemolysis through acquired pyrimidine 5'-nucleotidase (P5’N) deficiency [53, 54].

Both cadmium and lead have been related to cardiovascular outcomes [55–57]. It is posited that the most likely mechanism of action for these metals is that they compete with intracellular iron, increasing the amount of unbound iron ions that can accelerate the generation of reactive oxygen intermediates (ROI) through the super oxide/metal/hydrogen peroxide system [11, 58, 59]. Cadmium, for example, has been shown to generate intracellular ROI [11] and to be related to alterations in the antioxidant defense system [60]. Generation of ROI may underlie the ability of cadmium to inhibit hypoxia-inducible factor (HIF-1) activity and EPO induction [11, 49]. HIF-1 plays a major role in preventing ischemic CVD [61]. In addition, the generation of ROI may induce oxidative hemolysis [62]. Lead can substitute for iron when ingested and be actively transported into the body [63, 64]. Lead affects the activity of enzymes responsible for maintenance of oxidative-reductivity balance increasing the activity of glutathione peroxidase and superoxide dismutases [65].

Given the potential of these metals to disrupting iron’s action, it is relevant that we observed greater associations of both blood cadmium and lead exposure with RDW in the presence of iron deficiency (S2 Table). However, the fact that these associations were present even in the absence of iron deficiency may strengthen the hypothesis that the predominant pathway is through the metals’ ability to increase hemolysis. For example, in rodent models, cadmium was also shown to induce hemolysis [7]. Another consideration is the possible additive or multiplicative effect of exposure to both cadmium and lead. It has been suggested that combined exposure to lead and cadmium may increase the bioavailability of lead ion to enzymes in the heme pathway [51] and there was a suggestion of this in our observations.

Chronic kidney disease (CKD) is known to increase the risk of anemia, usually related to EPO deficiency [66, 67]. Studies have also independently related RDW to kidney function tests [68] and to mortality in kidney transplant recipients and acute kidney injury patients [69, 70]. Additionally, cadmium and lead have been shown to affect the renal system [71, 72]. Associations of cadmium and lead exposure with RDW persisted after additional adjustment for CKD (defined as estimated glomerular filtration rate) (S3 Table); however, because CKD may be one pathway by which these exposures affect RDW, such adjustments are inappropriate for estimating the overall association of the exposures with RDW.

This study has some limitations. It is a cross-sectional study so we cannot propose a causal pathway. There is a lack of understanding of the timing of the physical change in blood cell structure as it relates to subcellular insult. For instance, cadmium could induce iron deficiency or, and cadmium’s positive association with RDW could result from iron deficiency. Because the molecular pathways are just beginning to be elucidated, there is uncertainty regarding the dose and time-dependent nature of the mechanisms by which cadmium and lead relate to iron absorption. However, if lead or cadmium affects the risk of iron-deficiency anemia, our analyses adjusting for this condition could be affected by collider bias and could explain the attenuation of cadmium-RDW results. Also, although we adjusted for a number of confounders, we may not have adjusted adequately for important confounders such as tobacco smoke. For example, we were not able to adjust for pack-years of smoking because of limited data. Finally, blood cadmium concentrations in our population were relatively low with a number of non-detectable levels, potentially biasing our analyses of this measure as a continuous variable; however, we observed similar results when we divided blood cadmium into quartiles. The limit of detection for lead decreased an order of magnitude from the 2002–2011 cycles in the 2012–2016 cycles. Survey cycle was a significant confounder in the lead-RDW analyses possibly indicating a difference in the relationship of lead to RDW at very low lead levels or/and that the strongest influence of lead occurs at higher levels.

Strength of this study include that the association between environmental cadmium and lead on RDW was determined in a representative sample of U.S. population. Also, because of the relatively large sample size and wealth of data, we were able to test the associations stratified by sex, MCV category and smoking status.

## Conclusions

Our study provides evidence of a relationship between cadmium and RDW and between lead and RDW in the U.S. adult population. These results are consistent with and build upon animal and high exposure studies linking cadmium and lead exposure to hemolysis and erythropoietic disruption. Our results may shed light on differences in response with similar exposure. For example, the relationship of blood cadmium and lead to RDW was amplified in women and those with low MCV. Although prospective studies are needed to confirm the findings, our study underscores the need to address environmental exposures to cadmium and lead in the general population and in especially affected groups.

## Supporting information

S1 Fig Flow chart of study population for main and sub-analyses. (TIF)

S1 Table Multivariable-adjusted odds ratio for high red cell distribution width (RDW) for two-fold increases in blood cadmium and blood lead exposures, analyzed separately and together. (DOCX)

S2 Table Multivariable-adjusted differences in red cell distribution width (RDW) for two-fold increases in blood cadmium and blood lead exposures in main model and stratified by iron deficiency. (DOCX)

S3 Table Multivariable-adjusted differences in red cell distribution width (RDW) for two-fold increases in blood cadmium and blood lead exposures in main model and additionally controlling for chronic kidney disease (CKD). (DOCX)

## References

[1] Genchi G, Sinicropi MS, Lauria G, Carocci A, Catalano A. The effects of cadmium toxicity. Int J Environ Res Public Health. 2020;17(11). 10.3390/ijerph17113782 32466586 PMC7312803

[2] Sugawara E, Nakamura K, Fukumura A, Seki Y. Uptake of lead by human red blood cells and intracellular distribution. Kitasato Arch Exp Med. 1990;63(4):15–23. 2130186

[3] Goyer RA. Mechanisms of lead and cadmium nephrotoxicity. Toxicol Lett. 1989;46(1–3):153–62. 10.1016/0378-4274(89)90124-0 2650022

[4] Lacombe C, Dasilva JL, Bruneval P, Casadevall N, Camilleri JP, Bariety J, et al Erythropoietin—sites of synthesis and regulation of secretion. Am J Kidney Dis. 1991;18(4):14–9. 1928074

[5] Agency for Toxic Substances and Disease Registry (ATSDR). Toxicological profile for lead. Atlanta, GA: US Department of Health and Human Services, Public Health Service; 2007.

[6] Agency for Toxic Substances and Disease Registry (ATSDR). Toxicological profile for cadmium. Atlanta, GA: US Department of Health and Human Services, Public Health Service; 2012. 24049863

[7] Horiguchi H, Oguma E, Kayama F. Cadmium induces anemia through interdependent progress of hemolysis, body iron accumulation, and insufficient erythropoietin production in rats. Toxicol Sci. 2011;122(1):198–210. 10.1093/toxsci/kfr100 21540277

[8] Bressler JP, Olivi L, Cheong JH, Kim Y, Bannona D. Divalent metal transporter 1 in lead and cadmium transport. Ann N Y Acad Sci. 2004;1012:142–52. 10.1196/annals.1306.011 15105261

[9] Sanchez JR, Lynch DT. Histology, basophilic stippling. StatPearls. Treasure Island (FL)2020 31424843

[10] George JW, Duncan JR. The hematology of lead poisoning in man and animals. Vet Clin Pathol. 1979;8(1):23–30. 10.1111/j.1939-165x.1979.tb00878.x 15314779

[11] Horiguchi H, Kayama F, Oguma E, Willmore WG, Hradecky P, Bunn HF. Cadmium and platinum suppression of erythropoietin production in cell culture: clinical implications. Blood. 2000;96(12):3743–7. 11090055

[12] Thompson WG, Meola T, Lipkin M Jr., Freedman ML. Red cell distribution width, mean corpuscular volume, and transferrin saturation in the diagnosis of iron deficiency. Arch Intern Med. 1988;148(10):2128–30. 3178371

[13] Rai D, Wilson AM, Moosavi L. Histology, reticulocytes. StatPearls. Treasure Island (FL)2020 31194329

[14] Mohanty JG, Nagababu E, Rifkind JM. Red blood cell oxidative stress impairs oxygen delivery and induces red blood cell aging. Front Physiol. 2014;5:84 10.3389/fphys.2014.00084 24616707 PMC3937982

[15] Straat M, van Bruggen R, de Korte D, Juffermans NP. Red blood cell clearance in inflammation. Transfus Med Hemother. 2012;39(5):353–61. 10.1159/000342229 23801928 PMC3678279

[16] Pierce CN, Larson DF. Inflammatory cytokine inhibition of erythropoiesis in patients implanted with a mechanical circulatory assist device. Perfusion. 2005;20(2):83–90. 10.1191/0267659105pf793oa 15918445

[17] Semba RD, Patel KV, Ferrucci L, Sun K, Roy CN, Guralnik JM, et al Serum antioxidants and inflammation predict red cell distribution width in older women: the Women's Health and Aging Study I. Clin Nutr. 2010;29(5):600–4. 10.1016/j.clnu.2010.03.001 20334961 PMC3243048

[18] Ellingsen TS, Lappegard J, Skjelbakken T, Braekkan SK, Hansen JB. Red cell distribution width is associated with incident venous thromboembolism (VTE) and case-fatality after VTE in a general population. Thromb Haemost. 2015;113(1):193–200. 10.1160/TH14-04-0335 25274492

[19] Skjelbakken T, Lappegard J, Ellingsen TS, Barrett-Connor E, Brox J, Lochen ML, et al Red cell distribution width is associated with incident myocardial infarction in a general population: the Tromso Study. J Am Heart Assoc. 2014;3(4). 10.1161/JAHA.114.001109 PMC4310408 25134681

[20] Patel KV, Semba RD, Ferrucci L, Newman AB, Fried LP, Wallace RB, et al Red cell distribution width and mortality in older adults: a meta-analysis. J Gerontol A Biol Sci Med Sci. 2010;65(3):258–65. 10.1093/gerona/glp163 19880817 PMC2822283

[21] Zalawadiya SK, Veeranna V, Panaich SS, Afonso L. Red cell distribution width and risk of peripheral artery disease: analysis of National Health and Nutrition Examination Survey 1999–2004. Vasc Med. 2012;17(3):155–63. 10.1177/1358863X12442443 22615191

[22] Arbel Y, Weitzman D, Raz R, Steinvil A, Zeltser D, Berliner S, et al Red blood cell distribution width and the risk of cardiovascular morbidity and all-cause mortality. A population-based study. Thromb Haemost. 2014;111(2):300–7. 10.1160/TH13-07-0567 24173039

[23] Chen PC, Sung FC, Chien KL, Hsu HC, Su TC, Lee YT. Red blood cell distribution width and risk of cardiovascular events and mortality in a community cohort in Taiwan. Am J Epidemiol. 2010;171(2):214–20. 10.1093/aje/kwp360 20008450

[24] Tajuddin SM, Nalls MA, Zonderman AB, Evans MK. Association of red cell distribution width with all-cause and cardiovascular-specific mortality in African American and white adults: a prospective cohort study. J Transl Med. 2017;15(1):208 10.1186/s12967-017-1313-6 29029617 PMC5640961

[25] Pilling LC, Atkins JL, Kuchel GA, Ferrucci L, Melzer D. Red cell distribution width and common disease onsets in 240,477 healthy volunteers followed for up to 9 years. PLoS One. 2018;13(9):e0203504 10.1371/journal.pone.0203504 30212481 PMC6136726

[26] Foy BH, Carlson JCT, Reinertsen E, Padros I Valls R, Pallares Lopez R, Palanques-Tost E, et al Association of red blood cell distribution width with mortality risk in hospitalized adults with SARS-CoV-2 infection. JAMA Netw Open. 2020;3(9):e2022058 10.1001/jamanetworkopen.2020.22058 32965501 PMC7512057

[27] Salvagno GL, Sanchis-Gomar F, Picanza A, Lippi G. Red blood cell distribution width: a simple parameter with multiple clinical applications. Crit Rev Clin Lab Sci. 2015;52(2):86–105. 10.3109/10408363.2014.992064 25535770

[28] Lippi G, Mattiuzzi C, Cervellin G. Learning more and spending less with neglected laboratory parameters: the paradigmatic case of red blood cell distribution width. Acta Biomed. 2017;87(3):323–8. PMC10521889 28112703

[29] Li N, Zhou H, Tang Q. Red blood cell distribution width: a novel predictive indicator for cardiovascular and cerebrovascular diseases. Dis Markers. 2017;2017:7089493 10.1155/2017/7089493 29038615 PMC5606102

[30] Horne BD. A changing focus on the red cell distribution width: why does it predict mortality and other adverse medical outcomes? Cardiology. 2012;122(4):213–5. 10.1159/000341244 22890371

[31] Katsaros M, Paschos P, Giouleme O. Red cell distribution width as a marker of activity in inflammatory bowel disease: a narrative review. Ann Gastroenterol. 2020;33(4):348–54. 10.20524/aog.2020.0486 32624654 PMC7315702

[32] Aoki Y, Yee J, Mortensen ME. Blood cadmium by race/hispanic origin: the role of smoking. Environ Res. 2017;155:193–8. 10.1016/j.envres.2017.02.016 28231546 PMC5615218

[33] Loprinzi PD, Loenneke JP, Ahmed HM, Blaha MJ. Sex and race-ethnicity secular trends in mean and elevated red blood cell distribution width among adults in the United States, 1999–2012. Ethn Dis. 2016;26(1):45–50. 10.18865/ed.26.1.45 26843795 PMC4738854

[34] Patel KV, Harris TB, Faulhaber M, Angleman SB, Connelly S, Bauer DC, et al Racial variation in the relationship of anemia with mortality and mobility disability among older adults. Blood. 2007;109(11):4663–70. 10.1182/blood-2006-10-055384 17284526 PMC1885520

[35] Moubarac JC. Persisting problems related to race and ethnicity in public health and epidemiology research. Rev Saude Publica. 2013;47(1):104–15. 10.1590/s0034-89102013000100014 23703136

[36] Dugdale AE, Badrick T. Red blood cell distribution width (RDW)—a mechanism for normal variation and changes in pathological states. J Lab Precis Med. 2018;3.

[37] Camaschella C. Iron-deficiency anemia. N Engl J Med. 2015;372(19):1832–43. 10.1056/NEJMra1401038 25946282

[38] Beyazit Y, Kekilli M, Ibis M, Kurt M, Sayilir A, Onal IK, et al Can red cell distribution width help to discriminate benign from malignant biliary obstruction? A retrospective single center analysis. Hepatogastroenterology. 2012;59(117):1469–73. 10.5754/hge10676 22683963

[39] Karabulut A, Uyarel H, Uzunlar B, Cakmak M. Elevated red cell distribution width level predicts worse postinterventional thrombolysis in myocardial infarction flow reflecting abnormal reperfusion in acute myocardial infarction treated with a primary coronary intervention. Coron Artery Dis. 2012;23(1):68–72. 10.1097/MCA.0b013e32834f1188 22167053

[40] Turcato G, Serafini V, Dilda A, Bovo C, Caruso B, Ricci G, et al Red blood cell distribution width independently predicts medium-term mortality and major adverse cardiac events after an acute coronary syndrome. Ann Transl Med. 2016;4(13):5 10.21037/atm.2016.06.35 27500155 PMC4958725

[41] Saxena S, Weiner JM, Carmel R. Red blood cell distribution width in untreated pernicious anemia. Am J Clin Pathol. 1988;89(5):660–3. 10.1093/ajcp/89.5.660 3358371

[42] Vajpayee N, Graham SS, Bem S. Basic examination of blood and bone marrow In: McPherson RA, Pincus MR, editors. Henry's clinical diagnosis and management by laboratory methods. 22nd ed. Philadelphia: Elsevier/Saunders; 2011.

[43] Peters JL, Perlstein TS, Perry MJ, McNeely E, Weuve J. Cadmium exposure in association with history of stroke and heart failure. Environ Res. 2010;110(2):199–206. 10.1016/j.envres.2009.12.004 20060521 PMC3031174

[44] Tsaih SW, Korrick S, Schwartz J, Lee ML, Amarasiriwardena C, Aro A, et al Influence of bone resorption on the mobilization of lead from bone among middle-aged and elderly men: the Normative Aging Study. Environ Health Perspect. 2001;109(10):995–9. 10.1289/ehp.01109995 11675263 PMC1242074

[45] Peters JL, Kubzansky L, McNeely E, Schwartz J, Spiro A 3rd, Sparrow D, et al Stress as a potential modifier of the impact of lead levels on blood pressure: the Normative Aging Study. Environ Health Perspect. 2007;115(8):1154–9. 10.1289/ehp.10002 17687441 PMC1940093

[46] Perlstein TS, Weuve J, Pfeffer MA, Beckman JA. Red blood cell distribution width and mortality risk in a community-based prospective cohort. Arch Intern Med. 2009;169(6):588–94. 10.1001/archinternmed.2009.55 19307522 PMC3387573

[47] Friberg L, Piscator M, G. N. Cadmium in the environment. Cleveland: Chemical Rubber Company Press; 1971.

[48] Horiguchi H, Teranishi H, Niiya K, Aoshima K, Katoh T, Sakuragawa N, et al Hypoproduction of erythropoietin contributes to anemia in chronic cadmium intoxication: clinical study on Itai-itai disease in Japan. Arch Toxicol. 1994;68(10):632–6. 10.1007/BF03208342 7857202

[49] Chun YS, Choi E, Kim GT, Choi H, Kim CH, Lee MJ, et al Cadmium blocks hypoxia-inducible factor (HIF)-1-mediated response to hypoxia by stimulating the proteasome-dependent degradation of HIF-1alpha. Eur J Biochem. 2000;267(13):4198–204. 10.1046/j.1432-1327.2000.01453.x 10866824

[50] Paglia DE, Valentine WN, Dahlgren JG. Effects of low-level lead exposure on pyrimidine 5'-nucleotidase and other erythrocyte enzymes. Possible role of pyrimidine 5'-nucleotidase in the pathogenesis of lead-induced anemia. J Clin Invest. 1975;56(5):1164–9. 10.1172/JCI108192 1184742 PMC301979

[51] Madden EF, Fowler BA. Mechanisms of nephrotoxicity from metal combinations: a review. Drug Chem Toxicol. 2000;23(1):1–12. 10.1081/dct-100100098 10711385

[52] Pires JB, Miekeley N, Donangelo CM. Calcium supplementation during lactation blunts erythrocyte lead levels and delta-aminolevulinic acid dehydratase zinc-reactivation in women non-exposed to lead and with marginal calcium intakes. Toxicology. 2002;175(1–3):247–55. 10.1016/s0300-483x(02)00091-4 12049852

[53] Rees DC, Duley JA, Marinaki AM. Pyrimidine 5' nucleotidase deficiency. Br J Haematol. 2003;120(3):375–83. 10.1046/j.1365-2141.2003.03980.x 12580951

[54] Valentine WN, Paglia DE, Fink K, Madokoro G. Lead poisoning: association with hemolytic anemia, basophilic stippling, erythrocyte pyrimidine 5'-nucleotidase deficiency, and intraerythrocytic accumulation of pyrimidines. J Clin Invest. 1976;58(4):926–32. 10.1172/JCI108545 965496 PMC333255

[55] Tellez-Plaza M, Jones MR, Dominguez-Lucas A, Guallar E, Navas-Acien A. Cadmium exposure and clinical cardiovascular disease: a systematic review. Curr Atheroscler Rep. 2013;15(10):356 10.1007/s11883-013-0356-2 23955722 PMC3858820

[56] Navas-Acien A, Guallar E, Silbergeld EK, Rothenberg SJ. Lead exposure and cardiovascular disease—a systematic review. Environ Health Perspect. 2007;115(3):472–82. 10.1289/ehp.9785 17431501 PMC1849948

[57] Chowdhury R, Ramond A, O'Keeffe LM, Shahzad S, Kunutsor SK, Muka T, et al Environmental toxic metal contaminants and risk of cardiovascular disease: systematic review and meta-analysis. BMJ. 2018;362:k3310 10.1136/bmj.k3310 30158148 PMC6113772

[58] Ercal N, Gurer-Orhan H, Aykin-Burns N. Toxic metals and oxidative stress part I: mechanisms involved in metal-induced oxidative damage. Curr Top Med Chem. 2001;1(6):529–39. 10.2174/1568026013394831 11895129

[59] Jomova K, Valko M. Advances in metal-induced oxidative stress and human disease. Toxicology. 2011;283(2–3):65–87. 10.1016/j.tox.2011.03.001 21414382

[60] Messaoudi I, El Heni J, Hammouda F, Said K, Kerkeni A. Protective effects of selenium, zinc, or their combination on cadmium-induced oxidative stress in rat kidney. Biol Trace Elem Res. 2009;130(2):152–61. 10.1007/s12011-009-8324-y 19214400

[61] Semenza GL. Hypoxia-inducible factor 1 and cardiovascular disease. Annu Rev Physiol. 2014;76:39–56. 10.1146/annurev-physiol-021113-170322 23988176 PMC4696033

[62] Fibach E, Rachmilewitz E. The role of oxidative stress in hemolytic anemia. Curr Mol Med. 2008;8(7):609–19. 10.2174/156652408786241384 18991647

[63] Bradman A, Eskenazi B, Sutton P, Athanasoulis M, Goldman LR. Iron deficiency associated with higher blood lead in children living in contaminated environments. Environ Health Perspect. 2001;109(10):1079–84. 10.1289/ehp.011091079 11675273 PMC1242086

[64] Wright RO, Tsaih SW, Schwartz J, Wright RJ, Hu H. Association between iron deficiency and blood lead level in a longitudinal analysis of children followed in an urban primary care clinic. J Pediatr. 2003;142(1):9–14. 10.1067/mpd.2003.mpd0344 12520247

[65] Baranowska-Bosiacka I, Gutowska I, Marchlewicz M, Marchetti C, Kurzawski M, Dziedziejko V, et al Disrupted pro- and antioxidative balance as a mechanism of neurotoxicity induced by perinatal exposure to lead. Brain Res. 2012;1435:56–71. 10.1016/j.brainres.2011.11.062 22197700

[66] Astor BC, Muntner P, Levin A, Eustace JA, Coresh J. Association of kidney function with anemia: the Third National Health and Nutrition Examination Survey (1988–1994). Arch Intern Med. 2002;162(12):1401–8. 10.1001/archinte.162.12.1401 12076240

[67] Singh AK, Szczech L, Tang KL, Barnhart H, Sapp S, Wolfson M, et al Correction of anemia with epoetin alfa in chronic kidney disease. N Engl J Med. 2006;355(20):2085–98. 10.1056/NEJMoa065485 17108343

[68] Lippi G, Targher G, Montagnana M, Salvagno GL, Zoppini G, Guidi GC. Relationship between red blood cell distribution width and kidney function tests in a large cohort of unselected outpatients. Scand J Clin Lab Invest. 2008;68(8):745–8. 10.1080/00365510802213550 18618369

[69] Mucsi I, Ujszaszi A, Czira ME, Novak M, Molnar MZ. Red cell distribution width is associated with mortality in kidney transplant recipients. Int Urol Nephrol. 2014;46(3):641–51. 10.1007/s11255-013-0530-z 23959402

[70] Oh HJ, Park JT, Kim JK, Yoo DE, Kim SJ, Han SH, et al Red blood cell distribution width is an independent predictor of mortality in acute kidney injury patients treated with continuous renal replacement therapy. Nephrol Dial Transplant. 2012;27(2):589–94. 10.1093/ndt/gfr307 21712489

[71] Johri N, Jacquillet G, Unwin R. Heavy metal poisoning: the effects of cadmium on the kidney. Biometals. 2010;23(5):783–92. 10.1007/s10534-010-9328-y 20354761

[72] Loghman-Adham M. Renal effects of environmental and occupational lead exposure. Environ Health Perspect. 1997;105(9):928–38. 10.1289/ehp.97105928 9300927 PMC1470371

