# Supplementary figures and images for: The association of cadmium and lead exposures with red cell distribution width
Source: PLoS One. 2021 Jan 11;16(1):e0245173. doi: 10.1371/journal.pone.0245173 (PMC7801027; doi:10.1371/journal.pone.0245173)

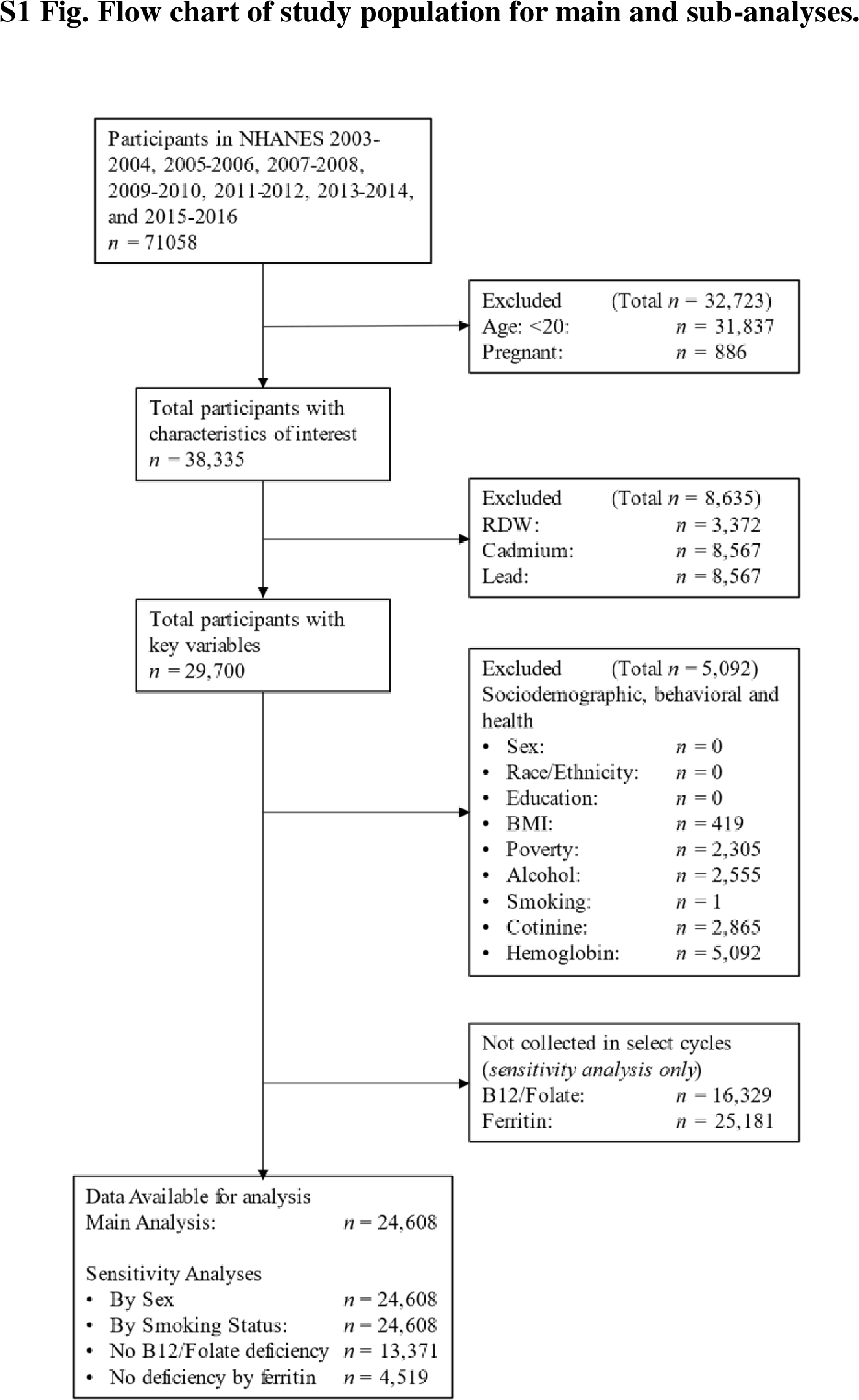

Supplement: S1 Fig — (TIF) [file pone.0245173.s001.tif]
