# Supplementary material for: The association of cadmium and lead exposures with red cell distribution width
Source: PLoS One. 2021 Jan 11;16(1):e0245173. doi: 10.1371/journal.pone.0245173 (PMC7801027; doi:10.1371/journal.pone.0245173)
Supplement: S1 Table — (DOCX) [file pone.0245173.s002.docx]

**S1 Table. Multivariable-adjusted odds ratio for high red cell distribution width (RDW) for two-fold increases in blood cadmium and blood lead exposures, analyzed separately and together.**

| Exposure | High RDW^a^  (Cases/Non-cases) | OR (95% CI) for high RDW per two-fold increase in heavy metal exposure |
| --- | --- | --- |
| Blood Cadmium |  |  |
| Main model^b^ | 1,688/22,919 | 1.67 (1.51, 1.84) |
| Further adjusted for iron  deficiency (ID)^c^ | 1,688/22,919 | 1.73 (1.56, 1.92) |
| Blood Lead |  |  |
| Main model^b^ | 1,688/22,919 | 1.10 (1.00, 1.21) |
| Further adjusted for ID^c^ | 1,688/22,919 | 1.21 (1.09, 1.34) |
| Both Blood Lead and Cadmium in Model |  |  |
| Main model^b^ |  |  |
| Blood Cadmium | 1,688/22,919 | 1.67 (1.52, 1.84) |
| Blood Lead | 1,688/22,919 | 0.99 (0.90, 1.09) |
| Further adjusted for ID^c^ |  |  |
| Blood Cadmium | 1,688/22,919 | 1.71 (1.54, 1.89) |
| Blood Lead | 1,688/22,919 | 1.08 (0.97, 1.20) |

^a^High RDW defined as RDW > 14.8%.

^b^Adjusted for age, sex, race/ethnicity, education, poverty income ratio, body mass index, alcohol consumption, smoking status, serum cotinine, and survey cycle.

^c^Adjusted for all variables in the main multivariable-adjusted model, and in addition, iron deficiency.
