# Supplementary material for: The association of cadmium and lead exposures with red cell distribution width
Source: PLoS One. 2021 Jan 11;16(1):e0245173. doi: 10.1371/journal.pone.0245173 (PMC7801027; doi:10.1371/journal.pone.0245173)
Supplement: S2 Table — (DOCX) [file pone.0245173.s003.docx]

**S2 Table. Multivariable-adjusted differences in red cell distribution width (RDW) for two-fold increases in blood cadmium and blood lead exposures in main model and stratified by iron deficiency.**

|  |  | **Mean difference in RDW, % (95% CI) per doubling of blood metal concentration** | | | | |
| --- | --- | --- | --- | --- | --- | --- |
|  | N | Cadmium | |  | Lead | |
| All, main model^a^ | 24,608 | 0.16 | (0.14, 0.18) |  | 0.04 | (0.01, 0.06) |
| Iron deficiency (ID)^b^, main model | 2,145 | 0.65 | (0.51, 0.78) |  | 0.21 | (0.05, 0.38) |
| Without ID, main model | 12,463 | 0.09 | (0.12, 0.12) |  | 0.07 | (0.05, 0.09) |

^a^Adjusted for age, sex, race/ethnicity, education, poverty income ratio, body mass index, alcohol consumption, smoking status, serum cotinine, and survey cycle.

^b^Iron deficiency is defined as hemoglobin <13 g/dL in men and hemoglobin <12 g/dL in women.
