# Supplementary material for: The association of cadmium and lead exposures with red cell distribution width
Source: PLoS One. 2021 Jan 11;16(1):e0245173. doi: 10.1371/journal.pone.0245173 (PMC7801027; doi:10.1371/journal.pone.0245173)
Supplement: S3 Table — (DOCX) [file pone.0245173.s004.docx]

**S3 Table. Multivariable-adjusted differences in red cell distribution width (RDW) for two-fold increases in blood cadmium and blood lead exposures in main model and additionally controlling for chronic kidney disease (CKD).**

|  | | |  | **Mean difference in RDW, % (95% CI) per doubling of blood metal concentration** | | | | |
| --- | --- | --- | --- | --- | --- | --- | --- | --- |
|  | | | N | Cadmium | |  | Lead | |
| Main model^a^ | | | 24,608 | 0.16 | (0.14, 0.18) |  | 0.04 | (0.01, 0.06) |
|  | Further adjusted for chronic kidney disease (CKD) ^b^ | | 24,473 | 0.12 | (0.11, 0.14) |  | 0.05 | (0.03, 0.08) |
|  | |  |  |  |  |  |  |  |

^b^Adjusted for age, sex, race/ethnicity, education, poverty income ratio, body mass index, alcohol consumption, smoking status, serum cotinine, and survey cycle.

^c^Adjusted for all variables in the main multivariable-adjusted model, and in addition, CKD.
